# *Hedyotis diffusa* Willd. Suppresses Hepatocellular Carcinoma via Downregulating AKT/mTOR Pathways

**DOI:** 10.1155/2021/5210152

**Published:** 2021-09-04

**Authors:** Lingli Huang, Hui Xu, Tianyu Wu, Gaofeng Li

**Affiliations:** ^1^Department of Oncology, Zhuzhou Hospital Affiliated to Xiangya School of Medicine, Central South University, Zhuzhou, China; ^2^Tongji Medical College, Huazhong University of Science and Technology, Wuhan 430030, China

## Abstract

**Objective:**

*Hedyotis diffusa* Willd. (HDW) is a famous Chinese herbal medicine, traditionally used to treat cancer in China. Currently, the clinically used drugs for the treatment of hepatocellular carcinoma (HCC) still have poor efficacy and have many side effects. HDW has fewer side effects after taking it, so this study explores the inhibitory effect of HDW on HCC, which may become a promising drug for the treatment of HCC.

**Methods:**

HCC cell lines such as SMMC-7721, SK-hep1, and Hep-G2 were treated with *Hedyotis diffusa* Willd. (HDW), after which migration was detected via transwell, while the proliferation of these cells was detected via MTT, CCK-8, and colony formation assays. Furthermore, protein levels were evaluated by western blotting, and Hep-G2 cells were implanted in nude mice to establish a xenograft model to evaluate the antitumor effect of the drug.

**Results:**

HDW exhibited the ability to inhibit the proliferation and migration of HCC cells. And its anticancer mechanism in hepatocellular carcinoma may be via AKT/mTOR pathway. Moreover, the drug use of HDW in the mouse model system has achieved a good effect. Importantly, it did not cause significant weight loss or hepatorenal toxicity.

**Conclusion:**

HDW can suppress the activation of the AKT/mTOR pathway in HCC cells, which may bring new light for the treatment of this kind of malignant tumor, but its exact mechanism still needs to be further explored.

## 1. Introduction

Hepatocellular carcinoma (HCC) is the sixth most common cancer in the world and the third most common cause of cancer death [1]. The current conventional treatment method is still surgical resection; the drug treatment method is still unsatisfactory; and the side effects of the currently used drugs are relatively large [2]. With the development of research, natural products and their structural analogs have made significant contributions to the drug treatment of cancer and are expected to provide a new path for drug treatment of cancer [3]. In a retrospective analysis of natural products used as the source of new drugs in the past four decades, it is shown that natural products are still the best choice for finding new drugs and can be effective drugs for the treatment of many human diseases [4]. Usually, the patients diagnosed with liver cancer miss the opportunity of operation, so it is urgent to seek alternative therapy for HCC.

*Hedyotis diffusa* Willd. (HDW) is a famous traditional Chinese herbal medicine with thousands of years of history. It is usually used to clear away heat, detoxify, and remove blood stasis, especially in the treatment of various types of inflammation [5]. Traditional Chinese medicine (TCM) has fewer side effects and has been clinically used to treat various diseases including cancer. Among them, HDW has long been used as an important ingredient in a variety of traditional Chinese medicine formulations to treat various types of cancer [6]. As an antitumor Chinese herbal medicine, it has been studied in the treatment of colorectal cancer [7, 8], breast cancer [9], and prostate cancer [10]. However, the effect of HDW on HCC and the potential mechanism of its antitumor effect have not been fully explored. A study based on network pharmacology found that the potential mechanism of HDW's anticancer may be due to its coordinated regulation of multiple cancer-related pathways, such as angiogenesis, cell differentiation, migration, apoptosis, invasion, and proliferation [10].

AKT/mTOR signaling pathway plays an important role in tumorigenesis, and abnormal AKT/mTOR pathways can be found in almost all cancers [11]. Overactivation of AKT/mTOR is associated with carcinogenesis and has become a potential molecular therapeutic target in cancer therapy. For example, trisubstituted imidazoles exert their anticancer activity through the PI3K/AKT/mTOR signaling pathway in breast cancer cells [12]. Moreover, mTOR is a drug target related to the development of cancer, and its inhibitors are usually used for the eradication of cancer and tumor stem cells [11]. The purpose of this study is to explore the antitumor effect of HDW on HCC, which may provide an effective alternative therapy for liver cancer.

## 2. Materials and Methods

### 2.1. Reagents

*Hedyotis diffusa* Willd. (S27202) was obtained from Shanghai Yuanye Bio-Technology Co. (Shanghai, China). Anti-*β*-actin, anti-AKT (Ser473), anti-mTOR (Ser2448), anti-MAPK (Erk1/2) (Thr202/Tyr204), anti-4EBP1 (Thr37/46), anti-Bcl-2, and anti-Bax were obtained from Cell Signaling Technology, MA, USA.

### 2.2. Preparation of *Hedyotis diffusa* Willd. (HDW)

A certain weight of HDW was weighed, after which PBS was added to form 20 mg/ml mother liquor, which was divided into multiple tubes and stored at −20°C. We took out one tube of mother liquor each time and diluted to the corresponding target concentration for the experiment.

### 2.3. Cell Culture

Human Hep-G2 HCC cells and SK-hep1 cells were provided by the Basic Medical College of Xiangya Medical College (Changsha, Hunan, China), while SMMC-7721 cells were provided by the Medical College of Hunan Normal University (Changsha, Hunan, China). Hep-G2 HCC cells and SMMC-7721 cells were grown in Dulbecco's modified Eagle's medium (HyClone, Logan, UT, USA), while SK-hep1 cells were grown in RPMI medium modified (HyClone, Logan, UT, USA). All these mediums contained 10% fetal bovine serum (FBS) and 1% penicillin/streptomycin (both from HyClone). And the culture temperature was 37°C, and the humidity was 5% CO_2_.

### 2.4. MTT Assay

MTT assay was used to evaluate cell viability. In short, cells were added to a 96-well plate (8 × 10^3^ cells/well) for 24 h, followed by a series of HDW concentrations for 72 h. Then MTT tetrazolium salt was added to each well (50 *μ*L; Sigma) for 5 h, after which 150 *μ*L of DMSO (Sigma) was added per well, and the absorbance was at 490 nm was evaluated via microplate reader (BioTek, Synergy HTX, VT, USA). SPSS 16.0 (IBM, IL, USA) was used to calculate the half-maximal inhibitory concentration (IC50) values from dose-response curves.

### 2.5. Cell Counting Kit (CCK-8) Assay

CCK-8 assay was also used to evaluate cell viability. According to the manufacturer's instructions, the CCK-8 Kit (AccuRef Scientific) was used to measure the survival rate of cells under the specified conditions. In short, cells were added to a 96-well plate (8 × 10^3^ cells/well) for 24 h, followed by a series of HDW concentrations for 72 h. Then, CCK-8 reagent was added to the wells, and the absorbance of each well at 450 nm was quantified using an automatic microplate reader (Bio-Rad, Hercules, CA, USA).

### 2.6. Colony Formation Assay

The ability of HDW to inhibit HCC cell proliferation was evaluated by colony formation assay. In short, cells were added to 24-well plates (8 × 10^3^ cells/well) for 24 h; then they were incubated with a range of HDW concentrations for another 5–7 days. Finally, cells were fixed with 10% formaldehyde, which were then stained with 0.1% crystal violet for 1 h at room temperature. Then absorbance at 550 nm was evaluated via a microplate reader.

### 2.7. Transwell Assay

Polycarbonate transwell filters were used to assess cellular migration. In short, 4 × 10^4^ cells in 200 *μ*L of serum-free medium were added to the upper chamber, while medium containing 10% FBS was placed in the lower chamber. After adding an appropriate amount of HDW solution for 24 hours, the upper chamber cells were removed, and the remaining cells were fixed with 10% formaldehyde for 30 minutes, stained with 0.1% crystallizer for 2 hours, and observed under a microscope.

### 2.8. siRNA Transfection

HCC cells were transfected with commercially AKT siRNA (Sense strand: 5ʹ-CGCCAUGGAUUACAAGUGUTT-3ʹ; Ribobio, China) or negative control (NC) siRNA (Sense strand: 5ʹ-UUCUCCGAACGUGUCACGUTT-3ʹ; Ribobio, China) with the transfection reagent Lipofectamine 2000 (Invitrogen); they were divided into siAKT group (AKT siRNA transfection) and siCtrl group (NC siRNA transfection) according to the different transfection siRNA. In short, cells were seeded in a six-well plate at a density of 3 × 10^5^ cells per culture dish, and cells at 30%–50% conflueny were first transfected with AKT siRNA or NC siRNA using Lipofectamine 2000 for 6 hours. After washing in PBS, the medium was replaced with a medium containing 10% serum for 36 hours. Finally, cell proteins are collected and confirmed by Western blot analysis to confirm specific silencing.

### 2.9. Western Blotting

The protein samples were separated by SDS-PAGE, transferred to membranes, and incubated with appropriate primary antibodies in a buffer containing bovine serum albumin (BSA) overnight at 4°C. The blots were then washed three times with PBS containing 0.1% Tween‐20 (PBST) for 15 minutes each time, stained with a secondary antibody for 1 h at room temperature, and then washed three times in PBST for 15 minutes each time. The protein bands were then detected with Pierce Super Signal chemiluminescent substrate (Rockford, IL) and imaged with a ChemiDoc system (Bio‐Rad). ImageJ (NIH, Bethesda, MD) was used for densitometric analyses; *β*‐actin was used for normalization; and protein expression was evaluated relative to the untreated control group.

### 2.10. Murine Xenograft Models

To evaluate HDW's antiproliferative effects on HCC tumors, female BALB/c-nu mice (4–6 weeks old) from Hunan SJA Laboratory Animal Co. Ltd. (Changsha, Hunan, China) were used to establish a xenograft nude mouse model. These experiments were consistent with the guidelines of the Institutional Animal Care and Use Committee at Hunan Normal University. Mice were housed under sterile conditions with access to food and water. A total of 12 mice were randomly selected, and 5 × 10^6^ Hep-G2 cells were subcutaneously implanted in their right flank. When the tumors grew to 50–80 mm^3^ in size, the mice were randomized into control group (100 *µ*l of 1% dimethyl sulfoxide (DMSO) plus 5% Tween and 5% neutral resin) and HDW (100 mg/kg/day) group (*n* = 6 animals/group). All treatments were performed in the stomach, and the administration was continued for 2 weeks. Tumor volumes and body weight were measured every two days, and tumor volume was calculated as follows: volume = 1/2 (length × width2). After two weeks, mice were euthanized with sodium pentobarbital, and tumor tissues were collected. The study was approved by the Institutional Research Ethics Committee of Xiangya Medical College (Hunan, China).

### 2.11. Histologic and Immunohistochemistry Analysis

After the animals were euthanized, the organisms including the liver and kidney were collected and fixed with 4% neutral-buffered formalin to prepare histologic slides. Then the samples were stained with hematoxylin and eosin (H&E), and 7-*μ*m tissue sections were analyzed via standard light microscopy (DFC450 C; Leica, Wetzlar, Germany). For immunohistochemical staining (IHC), antigen retrieval was performed by boiling in citrate antigen recovery solution (p0081; Beyotime) for 15 min. Then endogenous peroxidase activity was blocked by 3% H2O2. Ki67 (Abcam, ab15580, 1:200) antibody and a VECTASTAIN ABC kit (Vector Laboratories, Burlingame, CA) were used for staining, followed by the DAB substrate kit (Vector Laboratories) according to the manufacturer's instructions.

### 2.12. Statistical Analyses

Data were mean ± SD, and data were given with 95% confidence intervals and reported with corresponding *P*-values (^*∗*^*P* < 0.05, ^*∗∗*^*P* < 0.01, and ^*∗∗∗*^*P* < 0.001). The independent Student's *t-*test was used to analyze the significant differences between the two groups. For comparisons between multiple groups of samples, one-way analysis of variance (ANOVA) was used together with Tukey's multiple comparisons post hoc test unless otherwise specified. GraphPad Prism 6 and SPSS 13.0 were used for all statistical analyses.

## 3. Results

### 3.1. HDW Inhibits HCC Proliferation and Colony Formation Activity

**Figure 1:**
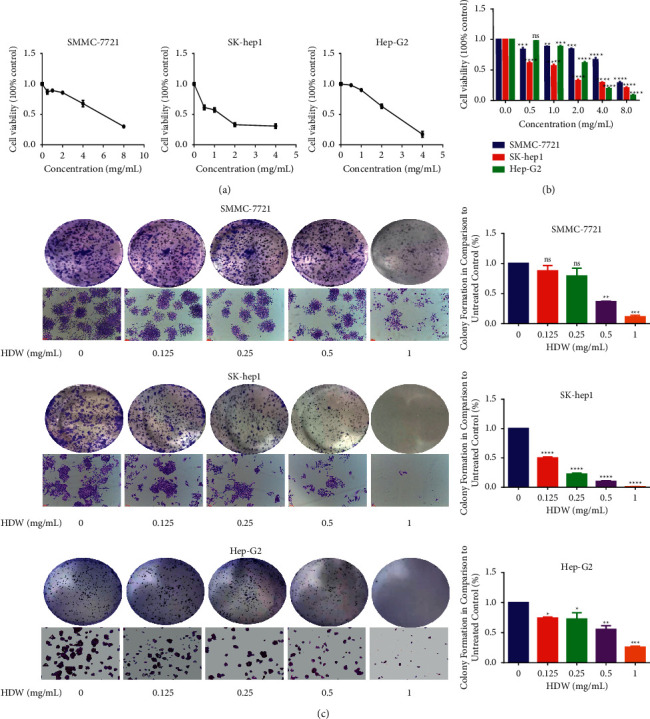
HDW affected the proliferation and colony-forming activity of SMMC-7721, SK-hep1, and Hep-G2 cells. (a, b) HDW suppressed the proliferation of SMMC-7721, SK-hep1, and Hep-G2 cells at 72 h post-HDW treatment via MTT assay and CCK-8 assay. (c) Conduct colony formation assay and image the wells at 550 nm after 5–7 days of HDW treatment. Data are means ± SD from triplicate experiments. ^*∗*^*P* < 0.05 and ^*∗∗*^*P* < 0.01, compared with the control (two-tailed *t*-test).

To evaluate the effect of HDW on the proliferation of HCC cells, SMMC-7721, SK-hep1, and Hep-G2 cells were treated with a series of HDW doses (Figure 1(a)), revealing a dose-dependent inhibition of the proliferation of the three cell types after 72 hours via MTT assay. The IC50 value is 5.045 mg/ml (SMMC-7721), 1.345 mg/ml (SK-hepG1), and 2.961 mg/ml (Hep-G2). Similarly, the CCK-8 experiment was used to detect the effects of different concentrations of HDW on the proliferative activity of three different liver cancer cell lines (Figure 1(b)). HDW also inhibited the colony formation of HCC cells in a dose-dependent manner, especially in Hep-G2 and SK-hepG1 cells (Figure 1(c)).

### 3.2. HDW Suppresses HCC Cell Migration

**Figure 2:**
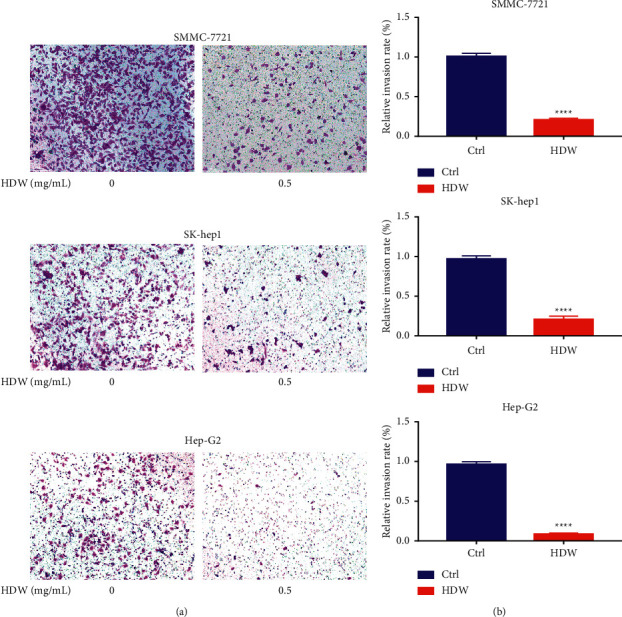
HDW impacted the migration of SMMC-7721, SK-hep1, and Hep-G2 cells. (a) HDW treatment suppressed the invasive activity of SMMC-7721, SK-hep1, and Hep-G2 cells in a transwell assay, with data being quantified in (b).

A transwell assay system was used to evaluate the ability of HDW to inhibit the migration of SMMC-7721, SK-hep1, and Hep-G2 cancer cells, which showed that the drug could inhibit the migration activity of HCC (Figures 2(a)–2(b)).

### 3.3. HDW Suppresses AKT/mTOR Pathway Activation

**Figure 3:**
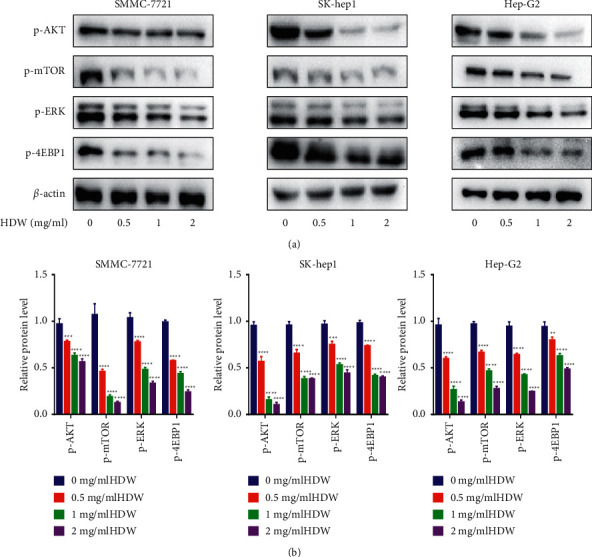
The effect of HDW on AKT/mTOR signaling in three HCC cell lines. The phosphorylated (P) protein levels in SMMC-7721, SK-hep1, and Hep-G2 cells were analyzed to evaluate how HDW affects signaling. (a) With *β*-actin as load control, the levels of P-AKT, P-mTOR, P-ERK, and P-4EBP1 were measured at 24 h post-HDW treatment. The control cells were untreated. (b) Relative protein levels (^*∗*^*P* < 0.05, ^*∗∗*^*P* < 0.01, and ^*∗∗∗*^*P* < 0.001; *n* = 3).

Next, western blotting was used to evaluate P-ERK, P-AKT, P-mTOR, and P-4EBP1 protein levels after HDW treatment in SMMC-7721 cells (0, 0.5, 1, and 2 ug/ml), SK-hep1 (0, 0.5, 1, and 2 ug/ml), and Hep-G2 cells (0, 0.5, 1, and 2 ug/ml; Figures 3(a)–3(b)). The results showed that HDW treatment downregulated the phosphorylation of all these proteins.

### 3.4. HDW Induces HCC Cells Apoptosis and Knockout of AKT Affects Cell Clone Formation

**Figure 4:**
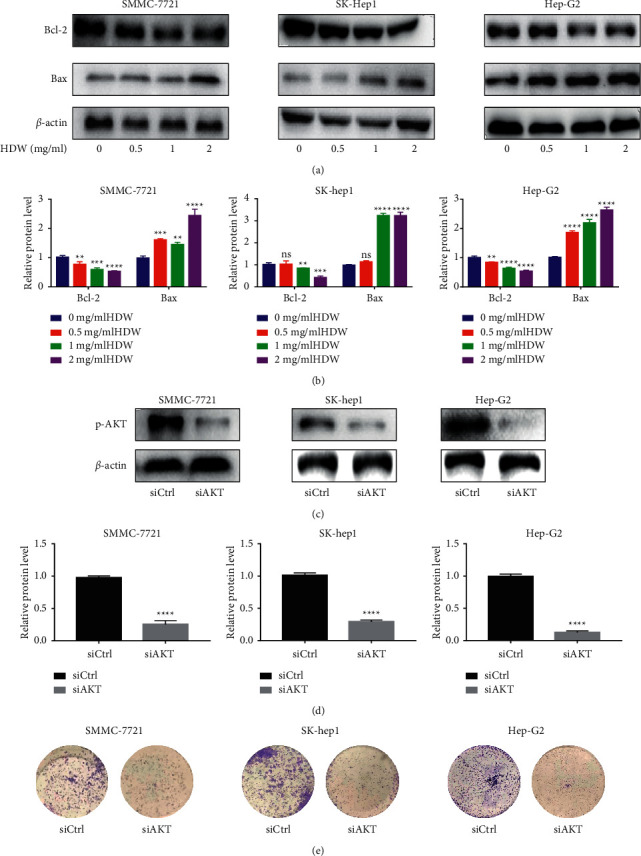
The effect of HDW on apoptosis in three different HCC cell lines and knockout of AKT affects cell clone formation. (a) With *β*-actin as load control, the apoptotic protein levels of Bcl-2 and Bax were measured at 24 h post-HDW treatment. The control cells were untreated. (b) Relative protein levels (^*∗*^*P* < 0.05, ^*∗∗*^*P* < 0.01, and ^*∗∗∗*^*P* < 0.001; *n* = 3). (c, d) The expression of p-AKT was detected by western blotting after transfection in three different HCC cell lines. (e) The effect of knockdown AKT on cell proliferation was evaluated using colony formation assay in SMMC-7721, SK-hep1, and Hep-G2 cells.

Three different HCC lines were treated with different concentrations of HDW (0, 0.5, 1, and 2 ug/ml) for 24 hours to detect the expression of apoptotic proteins Bcl-2 and Bax by western blotting (Figures 4(a) and 4(b)). These results strongly proved that HDW induced cell apoptosis. Subsequently, AKT was knocked out in the cells, and the transfection efficiency was verified by western blotting (Figures 4(c) and 4(d)). Furthermore, knockdown of AKT expression reduced colony formation of HCC cells (Figure 4(e)). These results found that knockdown of AKT expression significantly reduced the cell growth rate compared with the siCtrl group by the colony formation assay.

### 3.5. HDW Inhibits Xenograft HCC Tumor Growth in Mice

**Figure 5:**
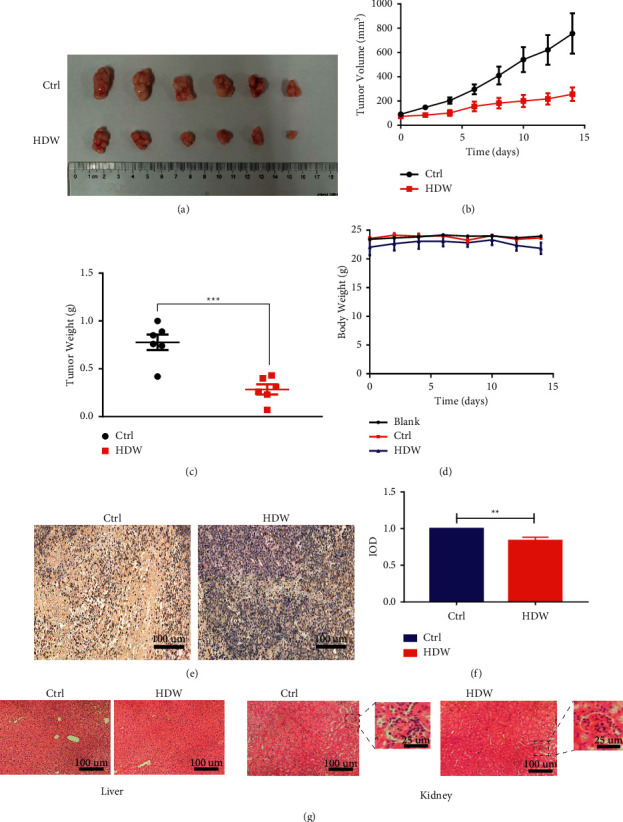
In vivo assessment of the effect of HDW on Hep-G2 xenograft tumor growth. (a) Tumor images. (b) Tumor volume changes. (c) Differences in tumor weight. (d) Murine body weight over time. (e) Immunohistochemistry images of staining of Ki67 in Hep-G2 cells of the xenografts injected with PBS or HDW. Scale bars, 100 *µ*m. (f) The relative values of IHC optical density. (g) H&E staining of the liver and kidney (^*∗*^*P* < 0.05, ^*∗∗*^*P* < 0.01, and ^*∗∗∗*^*P* < 0.001; *n* = 5).

To explore the efficacy of HDW treatment of HCC in vivo, nude mice were implanted subcutaneously with Hep-G2 cells, and then the mice were treated with the drug. Experiments showed that HDW treatment showed a good effect and significantly inhibited tumor growth (Figures 5(a) and 5(b)). Importantly, we observed a significant difference in tumor weight between the control group and the HDW treatment group (783 mg and 283 mg, respectively; *P* < 0.01; Figure 5(c)). And no significant weight loss was observed in any treatment group, which is in line with the safety of drug treatment (Figure 5(d)). Then the tumor suppressor effect of HDW was studied. Immunohistochemistry was performed on the xenograft. Compared with the control group, the cells of HDW-injected xenograft showed significantly lower Ki67 expression (Figures 5(e) and 5(f)). HDW treatment also did not cause any kidney or liver damage to treated mice (Figure 5(g)). All in all, these data indicate that HDW treatment in vivo can effectively inhibit the growth of HCC tumors.

## 4. Discussion

HCC is the fourth most deadly cancer and the fifth most common cancer in the world [13]. Although great efforts have been made to improve HCC treatment measures, the mortality rate of liver cancer patients is still very high, which is a serious threat to human life and health [14, 15]. As one of the common diseases that plague the whole world, HCC still has poor treatment effects [16]. At present, the commonly used therapeutic drugs have poor effects and large side effects, so it is urgent to find an alternative drug.

The development of cancer is strictly regulated by a variety of intracellular signaling pathways, such as AKT [17], mitogen-activated protein kinase (MAPK) [18], and STAT3 [19] pathways. The AKT signaling pathway is related to various types of cancer and is often related to anticancer therapy. When the AKT signal transduction pathway is activated, the mTOR signal transduction pathway is then activated, leading to abnormal cell proliferation, apoptosis, and differentiation [20]. These molecules play an important role in the induction, progression, and metastasis of HCC [21].

TCM has long been used to treat a variety of diseases, including cancer [22]. HDW is a natural plant extract, which has been used as a common ingredient in TCM for antibacterial and anti-inflammatory, enhancing immunity, antitumor, and other effects [23, 24]. With the progress of research, the anticancer effect of HDW has been paid more and more attention [25]. Therefore, this article studies the effect of HDW on HCC.

In this study, it was demonstrated that HDW reduced cell viability in different HCC cell lines in a dose-dependent manner. It is confirmed that HDW can inhibit cell proliferation and colony formation. It is worth noting that the downregulation of P-ERK, P-AKT, P-mTOR, and P-4EBP1 also provides further support, indicating that HDW can have an impact on HCC. HDW can exert anticancer activity through the AKT/mTOR pathway. It has also been observed that in vivo experiments HDW can significantly reduce the weight and volume of tumors and has low liver and kidney toxicity and side effects.

In short, HDW has good anticancer activity and few side effects. Therefore, HDW may be developed as a promising and potential anticancer drug for the clinical treatment of HCC.

## Data Availability

The data used to support the findings of this study are included within the article.
